# Oxford and Modern Medicine

**Published:** 1913-12-06

**Authors:** 


### OXFORD AND MODERN MEDICINE.

The proceedings at Oxford on the 26th ult. have decided us to deal, shortly, with the history of the earlier controversies at Oxford in regard to the progressive development of the Radcliffe Infirmary and the establishment in Oxford of a fully-equipped medical school. We may first remind our readers that these various matters are interestingly set forth and impartially dealt with in a monograph, "Oxford and Modern Medicine," by the late Sir Henry W. Acland, K.C.B., F.R.S., published by Henry Frowde in 1890. This publication consists of a letter by Sir Henry to Dr. James Andrew, then senior physician to St. Bartholomew's Hospital, and several valuable papers and facts, contained in a preface and appendices. It has been stated that Sir Henry Acland's views and action caused it to be erroneously felt by outsiders that he was largely responsible for the Medical School controversy during the period covered by the monograph. In fact, Sir Henry's heart was in his work and no one desired the Radcliffe Infirmary's welfare more. Sir Henry Acland was fully alive, as his statement on page 36 of the monograph shows, to the importance of helping to complete the organisation in the city of Oxford in health matters. For forty years he stated it to have been his great object to make the County Hospital a centre and type of medical work in the widest sense. Although the Radcliffe Infirmary was not typically constructed he held that it was good for study, seeing that it made a better subject for demonstration than the new St. Thomas's Hospital, because the former was full of mistakes, which had been fought against and partly conquered. He held that a reconstructed hospital is a fine centre for teaching purposes, seeing that the mistakes it has contained may be shown to be either the result, originally, of want of knowledge, or of opposition, or of narrow views and false economy. He maintained that directly the fullest knowledge exists in common public opinion of what constitutes perfection, and, when the necessary funds are forthcoming, there are experts, who, when wisely chosen, will so advise as to secure the maximum of completeness and efficiency.

Sir Henry shows that the opposition of his contemporaries, owing to the absence of knowledge in common hospital opinion, was so great and so persistent as to constitute instructive and sometimes startling reading for the initiated of to-day. Sir Henry Acland's main aim was to prevent Oxford medical graduates from being made into a class by themselves, because then they would be trained only for medical examinations, instead of providing that the strictly scientific parts of medical training may be gone through at Oxford in the best way, so that through Oxford members of the medical profession should be connected with liberal studies.

In brief, then, Sir Henry Acland's point of view was that, in addition to the degree in Arts, the medical student should study and pass at Oxford everything except the practical parts of the medical profession, which latter her sons must learn in the great London schools of medicine. Sir Henry has been justified in this view by what has happened since his day and by the state of university and medical opinion at the present time. All honour to Sir Henry Acland. One of his last efforts was made in March 1889, when he tried to bring before the governors of the Radcliffe Infirmary a motion constituting the Regius Professor of Medicine for the time being an *ex-officio* member of its board of management, and placing under his charge a fixed number of medical beds and cots for children. These regulations were to come into force on Sir Henry Acland's retirement. Instead of welcoming this proposal, the board of the hospital refused to place Sir Henry's motion on the agenda or to allow it to be even discussed. Such a board would ruin a Bank, let alone a Hospital.

